# Case Report: Flow cytometric differential diagnosis of a peripheral T-cell lymphoma, NOS with complete loss of CD45 and dim expression of CD3

**DOI:** 10.3389/pore.2025.1612095

**Published:** 2025-05-29

**Authors:** Gábor Szalóki, Ágota Szepesi, Ilona Tárkányi, Ágnes Márk, Csilla Kriston, Anna Hunyadi, Réka Mózes, Gábor Barna

**Affiliations:** ^1^ Department of Pathology and Experimental Cancer Research, Semmelweis University, Budapest, Hungary; ^2^ Department of Internal Medicine and Haematology, Faculty of Medicine, Semmelweis University, Budapest, Hungary

**Keywords:** flow cytometry, immunophenotype, peripheral T-cell lymphoma, differential diagnosis, PTCL, NOS

## Abstract

Peripheral T-cell lymphomas (PTCLs) are a group of non-Hodgkin lymphomas originating from mature T-lymphocytes. Despite encompassing several well-defined entities, about 25% of the PTCLs do not fulfill the requirements of any of the subcategories. These diseases are classified as PTCL, not otherwise specified (PTCL, NOS), and often associated with poor prognosis. Hereby we present a case of a female patient, diagnosed with PTCL, NOS from her skin biopsy specimen. Besides histology and immunohistochemistry, flow cytometry was used for phenotyping and staging (peripheral blood, bone marrow). Pathologic T-cells were found in all the investigated tissues, with a very unusual CD45 negative and surface CD3 dim immunophenotype. For proper differential diagnosis, we determined several markers with immunohistochemistry (CD3, CD4, CD7, CD8, CD30, PD1, Ki-67) and flow cytometry: (CD2, cytoplasmic CD3, surface CD3, CD4, CD5, CD7, CD8, CD9, CD10, CD19, CD20, CD26, CD34, CD38, CD45, CD48, CD56, CD99, CD123, surface TRBC1, cytosplasmic TRBC1, surface TRBC2, cytoplasmic TRBC2, MPO, TdT, Igκ, Igλ). Here we discuss the difficulties of the differential diagnostic process and highlight some potential pitfalls of flow cytometric analysis of the pathologic T-cells with such a rare immunophenotype. Despite several determined markers, the disease characteristics did not meet the criteria of any PTCL subtype, therefore the diagnosis remained PTCL, NOS. Due to the aggressive course of the disease, we lost the patient within 1 year after the diagnosis.

## Introduction

T-cell non-Hodgkin lymphomas (T-NHL), often called mature or peripheral T-cell lymphomas (PTCLs) are a heterogeneous group of non-Hodgkin lymphomas originating from post-thymic T-lymphocytes [1]. Despite the human immune system consisting of much more T-lymphocytes than B-cells, T-NHLs account for less than 15% of all non-Hodgkin lymphomas [2, 3]. Regardless of their minority among NHLs, PTCLs are classified into more than 30 entities according to the latest classification systems [4–6]. Most PTCLs show an aggressive clinical course and are challenging to manage with therapies that are effective in B-cell NHLs [7, 8]. Newly developed targeted and biological therapies can improve the outcome of some well-defined subtypes of PTCLs [8]. Proper pathological diagnosis and classification of PTCLs require complex analysis of the patient’s samples including morphology, immunophenotyping, and molecular genetics. Despite our increasing knowledge about PTCL subtypes, more than 25% of them do not fulfill the requirements of any entities and therefore are classified as peripheral T-cell lymphoma, not otherwise specified (PTCL, NOS) [2]. PTCL, NOS is a complex category, collecting clinically diverse and biologically heterogenous diseases, mostly associated with poor prognosis [6].

In the diagnosis and classification of PTCLs besides histology, immunohistochemistry, and molecular genetics, flow cytometry plays an important role in phenotyping. It is a powerful tool to detect the co-expression of several (even over 10) antigens, that can facilitate the classification of the disease. Several antigens are detected including lineage (CD45, CD19, CD20, CD3, CD4, CD8), pan-T-cell (CD2, CD5, CD7, CD8) and other (CD1a, CD10, CD25, CD30, CD34, CD56, CD57, CD279, CXCR5, TCRα/β, TCRγ/δ, TdT, TRBC1) markers [9–12]. Since there is no consensus among diagnostic flow cytometry laboratories about T-lymphocyte phenotyping panels, laboratories use different approaches to identify and type T-cells. This also means these panels have different strengths and weaknesses.

Hereby we present a PTCL, NOS case with a rare phenotype raising differential diagnostic problems. The malignant T-cells showed almost complete loss of CD3 and CD4 antigens and were negative for CD45. This unfortunate combination of surface marker loss makes the identification of the tumor cells difficult and raises questions about the classification of the disease. We will review this case from a differential diagnostic and methodological perspective.

### Case description

The first symptoms of a 49-year-old female patient born in 1975, were presented in January of 2024 as diffuse erythroderma accompanied by mild leukocytosis. Histology of her skin biopsy specimen revealed a dominantly dermal, perivascular T-lymphocytic infiltration showing multifocal epidermotropism. PCR confirmed T-cell monoclonality. A CT scan raised the possible involvement of some lymph nodes in the inguinal and parailiac regions. Flow cytometry, cytology, and immunohistochemistry confirmed peripheral blood, bone marrow, and an inguinal lymph node involvement. Based on the morphological features and the immunophenotype of the tumor cells the patient was diagnosed with peripheral T-cell lymphoma, not otherwise specified (PTCL, NOS).

Due to the rapid progression of the disease, the patient was treated with Adcetris-CHEP (brentuximab-vedotin, doxorubicin, etoposide, cyclophosphamide). Since the treatment turned out to be intolerable for the patient, she was further treated with gemcitabine and oxaliplatin (GemOx) supplemented with venetoclax. The patient received five cycles of GemOx therapy, but the 8th and 15th-day doses had to be canceled in every cycle due to thrombocytopenia. Despite this, the patient showed a satisfactory response, and the worsening of the skin symptoms decelerated. For her hardly tolerable skin symptoms, she received palliative radiotherapy on her extremities. Later, due to dermal relapse, she received ICE (ifosfamide, carboplatin, etoposide) treatment with radiotherapy.

**FIGURE 1:**
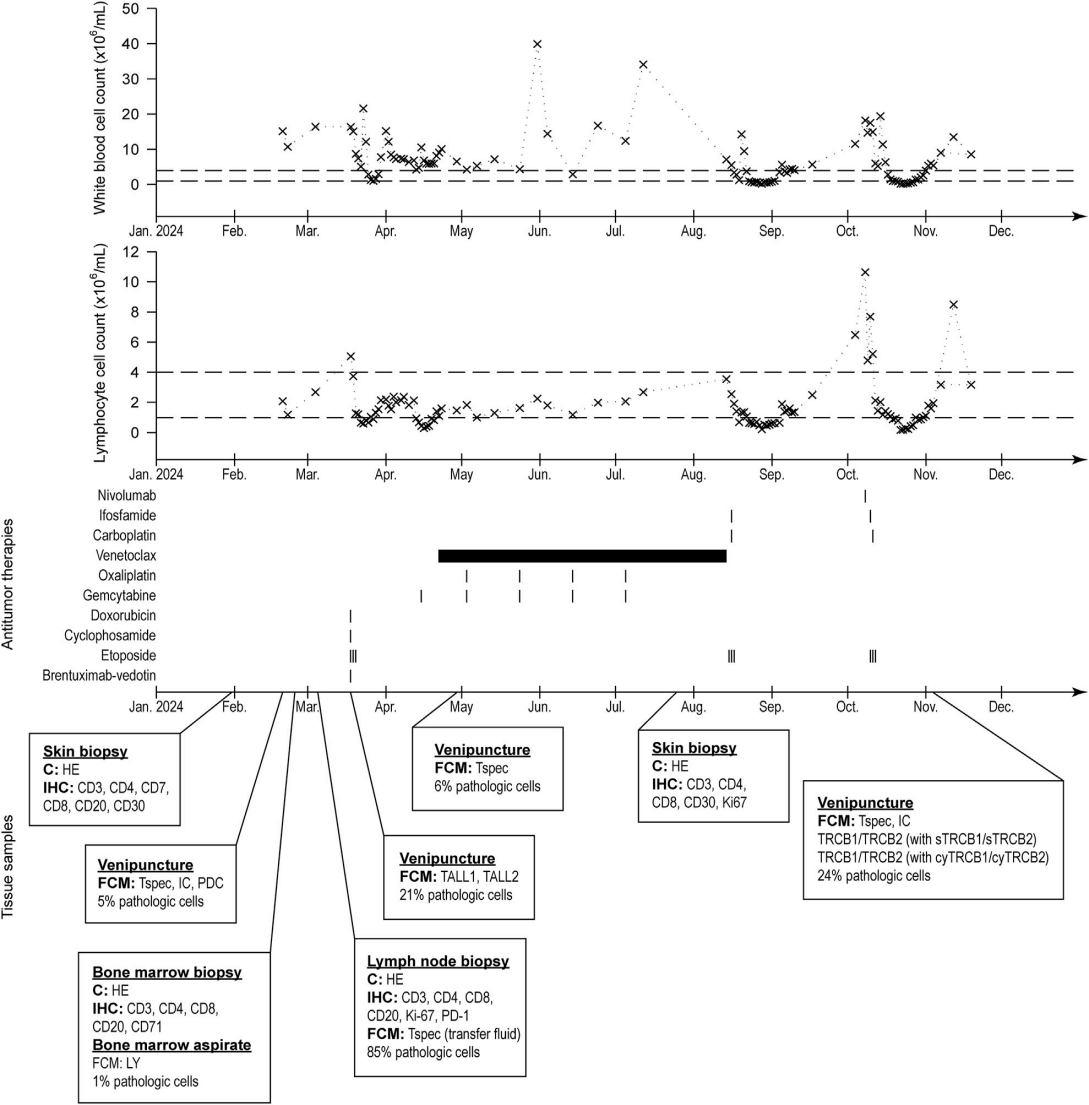
History of the patient’s white blood cell and lymphocyte count (normal ranges indicated with dashed lines), treatments, and collected samples for pathologic evaluation.

Despite the therapies and the efforts, the condition of the patient was constantly deteriorating, and she passed away 11 months after the diagnosis. Her detailed diagnostic and treatment history can be seen in Figure 1.

## Methods

### Preparation of patient samples for immunostaining and flow cytometry

Peripheral blood samples, bone marrow aspirate, and lymph node biopsy were immunophenotyped by flow cytometry. Peripheral blood samples did not require preparation; the bone marrow aspirate was filtered with a 40 µm mesh filter before staining. The lymph node sample was cut into small pieces with scissors in phosphate-buffered saline (PBS, 137 mM NaCl, 2.7 mM KCl, 10 mM Na_2_HPO_4_, 1.8 mM KH_2_PO_4_, pH = 7.4), vortexed vigorously and liberated cells were filtered with a 40 µm mesh filter.

We measured the following markers with flow cytometry: common leukocyte antigen: CD45; T-lymphoid lineage markers: cytoplasmic CD3 (cyCD3), surface CD3 (sCD3), surface TRBC1 (sTRBC1), cytoplasmic TRBC1 (cyTRBC1), surface TRBC2 (sTRBC2), cytoplasmic TRBC2 (cyTRBC2); other T-cell markers: CD2, CD4, CD5, CD7, CD8; B-lymphoid lineage markers: CD19, CD20, Igκ, Igλ; NK-cell lineage marker: CD56; myeloid lineage markers: MPO (cytoplasmic); immaturity markers: CD9, CD10, CD34, CD48, CD99, TdT (cytoplasmic); activation and aberrant markers: CD26, CD38, CD123. The panel descriptions are shown in Supplementary Table S1.

For surface staining 50 μL cell suspension (containing a maximum of 1 × 10^6^ cells) was labeled with an antibody mixture. The samples were incubated at 4°C in the dark for 15 min. Then 1 mL of FACSLysing Solution (BD Bioscience) red blood cell lysing solution was added to the samples and further incubated for 10 min, in the dark. Finally, we washed the samples two times (800 x g at room temperature for 5 min) with PBS and resuspended the resulting pellet in 300 µL PBS containing 33 nM SYTO 41 nuclear acid stain.

For intracellular staining 50 μL cell suspension (containing a maximum of 1 × 10^6^ cells) was labeled with a surface antibody mixture according to Supplementary Table S1. The samples were incubated at 4°C in the dark for 15 min. Then 100 µL Intrastain A solution (Beckman Coulter Life Sciences) was added and incubated for 15 min in the dark. Then the samples were washed in PBS 800 x g at room temperature for 5 min) and the pellet was resuspended in 100 µL Intrastain B solution containing the intracellular antibodies. After 15 min incubation in the dark, the samples were washed, and the resulting pellet was resuspended in 300 µL PBS containing 33 nM SYTO 41 nuclear acid stain.

The samples were measured using a 10-color BD FACSLyric (BD Biosciences, United States) flow cytometer routinely calibrated with BD CS&T beads (BD Biosciences). At least 50.000 events were acquired, and the data was analyzed with the Kaluza 2.2 software (Beckman Coulter, United States).

### Histology and immunohistochemistry

Immunohistochemistry was performed by a LEICA Bond Max (Leica Biosystems, Germany) system. For antigen localization, DAB polymer (Leica Biosystems) was used. Antibodies used for immunohistochemical staining were as follows: CD3 (polyclonal, Dako, Agilent Technologies), CD4 (1F6, Leica Biosystems), CD7 (LP15, Leica Biosystems), CD8 (EP34, Bio SB, USA), CD30 (Ber-H2, Dako, Agilent Technologies), Ki-67 (MIB1, Dako), PD1 (NAT105, Cell Marque), TCL1 (MRQ-7, Cell Marque). The slides were scanned with Panoramic^®^ 1000 DX scanner (3DHistech LTD., Hungary) and analyzed with the SlideViewer (3DHistech LTD.) software.

### T-cell receptor rearrangement testing

The rearrangement of TcR-β and TcR-γ genes was tested with polymerase chain reaction (PCR) using multiplex primer sets according to the BIOMED-2 guidelines [13, 14].

## Results

### Skin biopsies

The first diagnostic sample was a skin punch biopsy specimen that showed perivascular T-cells infiltrate, dominantly in the dermis, spreading in the epidermis. The pathological T-cells were positive for CD3 and CD7; showed partial expression of CD4, and were negative for CD8, and CD30 (Supplementary Figure S1).

A skin punch biopsy taken 5 months later confirmed the progression of the disease. Diffuse infiltration of the dermis was found with approximately 50% proliferation rate (measured by Ki-67 immunostaining, Supplementary Figure S2). TCL1 immunostaining of the second specimen was also negative (Supplementary Figures S2A–G).

### Testing T-cell receptor rearrangement

The molecular genetic examination of the formalin-fixed, and paraffin-embedded skin biopsy specimen revealed monoclonal rearrangement of the TCR beta and gamma genes (Supplementary Figures S1H,J).

### Blood samples

**FIGURE 2:**
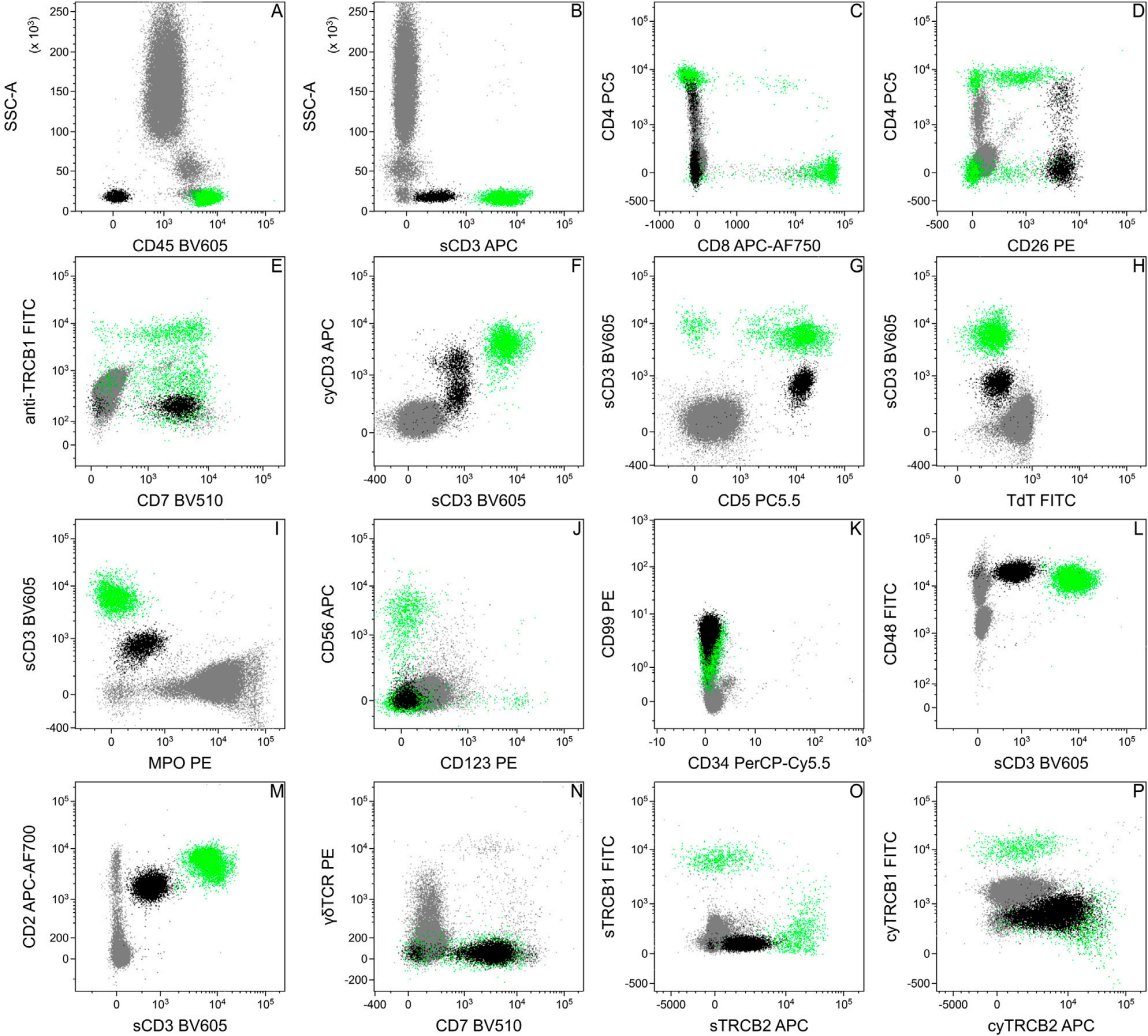
Flow cytometric analysis of the patient’s peripheral blood. Based on the flow cytometric data, the immunophenotype of the pathologic cells (black) was the following: positive: CD2 **(M)**, CD5 **(G)**, CD7 **(E, N)**, CD26 **(D)**, cyCD3 **(F)**, cyTRBC2 **(P)**; dim: sCD3 **(B,F,G,H,I,L,M)**, sTRBC2 **(O)**; heterogeneous: CD4 **(C)**; negative: CD8 **(C)**, CD45 **(A)**, CD56 **(J)**, CD123 **(J)**, sTRBC1 **(O)**, cyTRBC1 **(P)**, TdT **(H)**, MPO **(I)** and γδTCR **(N)**. The dot plots show data of peripheral blood from different time points, therefore the ratio of the pathological cells can be different. Normal T-lymphocytes for positive control were highlighted in green.

We tested the patient’s peripheral blood sample at our flow cytometry laboratory shortly after the skin biopsy specimen (Figure 1). The white blood cell count was slightly elevated (10.73 × 10^6^/mL), but the lymphocyte count was only 1.17 × 10^6^/mL. Flow cytometry analysis revealed 5% normal and 5% aberrant T-lymphocytes among all leukocytes in the sample. The immunophenotype was the following: the cells were positive for CD5, CD7, CD26, and cyCD3 (less positive than normal T-cells); dim for sCD3; heterogeneous for CD4 (mostly negative) and negative for CD8, CD45, CD56, CD123, sTRBC1, TdT, MPO (Figure 2). Later, investigating further blood samples the immunophenotype was completed with the following markers: positive for CD2 (slightly lower than normal T-cells), CD48, CD99 (like normal T-cells), and cyTRBC2; weak for sTRBC2 and negative for CD34, cyTRBC1, γδTCR. 9 months later the flow cytometry of the peripheral blood confirmed progression with a 24% tumor cell ratio (Figure 2).

### Bone marrow biopsy and aspirate

Histology and immunohistochemistry of the bone marrow biopsy revealed one cluster of infiltrating T-cells containing dominantly CD4 and CD3-positive cells (Supplementary Figure S3). Flow cytometry of the bone marrow aspirate found one percent infiltrating pathological T-cells. The immunophenotype of the pathological T-cells in the bone marrow and in the peripheral blood was identical (Supplementary Figure S4) and was negative for B-cell markers: CD19, CD20, Igκ, and Igλ (data not shown).

### Inguinal tumor biopsy

A core biopsy was carried out of an inguinal lymph node. Both histology (Supplementary Figure S5) and flow cytometry (Supplementary Figure S6) found massive infiltration (over 85%) of pathological T-lymphocytes. The immunophenotype corresponded to the previous findings, immunohistochemistry revealed PD1 negativity and more than 50% Ki-67 positive ratio of the pathological cells.

## Discussion

The presented T-cell lymphoma case raised several differential diagnostic questions based on the unusual clinical picture starting as a cutaneous process but developing systemic involvement and rapid progression. Flow cytometry is an important tool in the diagnostic process to clarify the exact phenotype and to detect small amounts of tumor cells during the staging in the different body compartments.

Here we focus on the flow cytometric differential diagnosis of the case, but we cannot ignore the histological and immunohistochemical results of the skin biopsy specimen. The primary findings based on the first skin biopsy referred to a mature T-cell lymphoma with CD7, CD3, and partially CD4 positive phenotype associated with mild epidermotropism and erythroderma (Supplementary Figure S1), that in the first place raised the diagnosis of a cutaneous T-cell lymphoma (e.g., mycosis fungoides, Sezary syndrome). Based on the histologic pattern and CD30 negativity (Supplementary Figures S1, S2.) anaplastic large cell lymphoma (ALTL) was excluded. During the staging procedure, pathologic T-cells were detected in the peripheral blood in small proportion by flow cytometry with a very unusual CD45 negative, sCD3 dim, and almost surface CD4 negative immunophenotype (Figures 2A,B), which changed the diagnostic process. Due to the loss of CD45, surface CD3, and CD4, the possibility of a precursor T-cell lymphoma had to be excluded. Although the skin involvement of T-lymphoblastic lymphoma is rare, there are some examples [15, 16]. Tembhare et al. [17] made a useful flowchart to differentiate between mature T-cell neoplasms based on their immunophenotype. Among others [5, 10], we used this flowchart as the basis of the differential diagnosis. Despite the low expression of sCD3, loss of CD45, and expression of cyCD3, neither other blast markers, e.g., presence of CD34, increased expression of CD99, nor loss of CD48 (Figures 2A,B,F,K,L), nor the histologic images supported the precursor T-cell origin of the pathological cells. Based on strong CD26 surface expression (Figure 2D) the diagnosis of mycosis fungoides, and Sezary syndrome, were excluded [18]. For the sake of safety CD56 and CD123 expressions were checked (Figure 2J) to test the blastic dendritic cell origin of tumor cells [19]. The negativity of both markers and the heterogeneous CD4 expression did not support the diagnosis of blastic dendritic cell neoplasm [19]. The negativity of CD10 (Supplementary Figure S4) and CD279 (PD1, Supplementary Figure S5) made the diagnosis of angioimmunoblastic T-cell lymphoma or follicular helper type T-cell lymphoma less likely. The diagnosis of any leukemic type mature T-cell lymphomas was also not supported since the blood involvement did not meet the diagnostic criteria [20, 21], although Tharkal et al. reported a T-cell prolymphocytic leukemia (T-PLL) with a similar immunophenotype [22], but without information of any skin involvement. In our case, the morphology of the pathologic cells, the skin involvement, the minimal peripheral (see the lymphocyte count in Figure 1) and bone marrow involvement (Supplementary Figure S4) made T-PLL unlikely. TCL1 negative immunostaining of the patient’s skin biopsy specimen also ruled out the diagnosis of T-PLL (Supplementary Figure S2).

Reduced expression of surface CD3 is an often-discussed phenomenon in the case of T-cell lymphomas. In contrast, the loss of CD45 is rarely mentioned. Studies that reviewed a large number of cases either did not talk about CD45 expression [23] or found only a few [10] or did not find [11] CD45 negative cases. Therefore, unexpected loss of CD45 of the T-cell lymphoma cells can complicate the diagnostic process, require extended differential diagnosis, and make the final decision difficult.

### Flow cytometric immunophenotyping pitfalls

Based on the findings of histology and immunohistochemistry we expected potential pathologic cells in the lymphocyte gate, with bright CD45 and positive surface CD3 expression. During the FCM analysis of blood, due to the loss of CD45, pathologic cells appeared in the erythroid gate on the CD45-SSC dot plot (Figure 2A), which created the opportunity to misinterpret these cells as erythroid or debris. This problem was more expressed in the case of bone marrow aspirate where we normally expected erythroid precursors (Supplementary Figure S4). The dim surface CD3 expression of the pathological cells also made their identification difficult, even on the sCD3-SSC dot plot, since their separation is incomplete from the erythroid cells or other CD45 negative cells (Supplementary Figures S4, S6).

**FIGURE 3:**
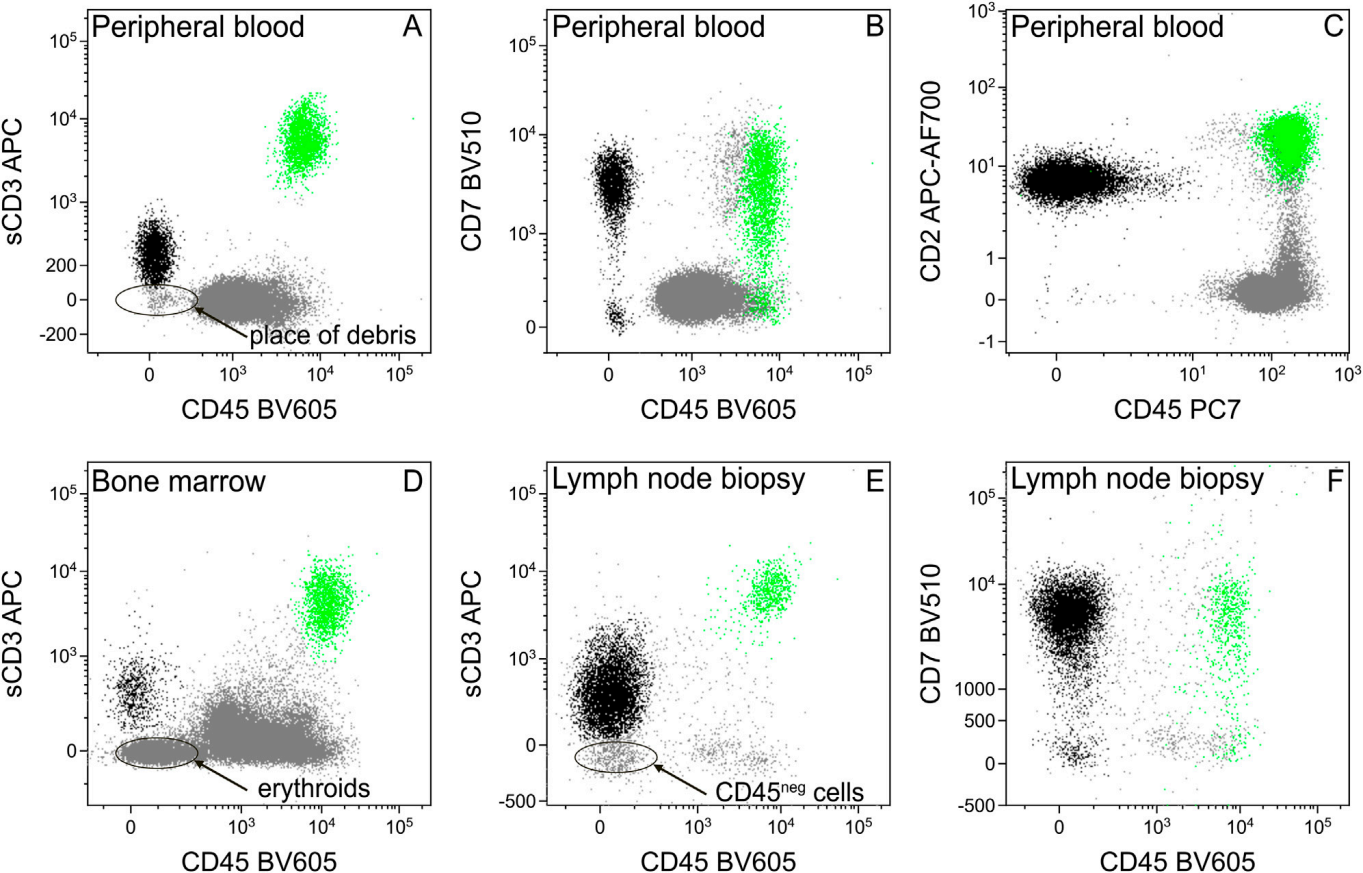
Separation of tumor cells from debris, erythroids, or other CD45 negative cells using different T-cell markers. The sCD3 dim tumor cells (black) cannot be separated clearly from the CD45 negative particles, like debris [in the case of peripheral blood, **(A)**], erythroid precursors [in the case of bone marrow, **(D)**], or other cells [in the case of hematopoietic tissues, e.g., lymph node, **(E)**]. Introducing other T-cell markers (CD7, CD2) facilitates the chance of successful separation **(B,C,F)**. Normal T-lymphocytes for positive control were highlighted in green.

A DNA dye (we used SYTO41) can serve well to differentiate between cells and debris. Introducing alternative gating strategies (here a sCD3-CD45 dot plot, Figures 3A,D,E) can facilitate the identification of aberrant cells, with unusual expression and, despite sCD3 being the most frequent marker to identify T-lymphocytes, adding other T-lymphoid markers (CD7, CD2) to the analysis can be also useful (Figures 3B,C,F).

Reduced expression of surface CD3 is an indicator of the disconcerted assembly of the T-cell receptor complex (TCR) on the cell surface. Therefore, to verify T-lymphoid lineage, cytoplasmic labeling of CD3 is a criterion (Figure 2F). Furthermore, due to reduced expression of TCR, clonality assessment by TRBC1 or TRBC2 labeling can be also unsuccessful. Luckily in our case, the pathological T-cell showed a reduced, but detectable expression of TRBC2 (Figure 2O). Besides, we could test clonality labeling intracytoplasmic TRBC1 and TRBC2. Strong expression of cyTRBC2 verified the monoclonal origin of the aberrant cells (Figure 2P).

## Conclusion

Peripheral T-cell lymphomas are a heterogeneous group of non-Hodgkin lymphomas originating from mature T-lymphocytes. Despite several cases that can be classified into one of the subtypes, there are ones that do not fulfill the criteria of any of them. These cases are diagnosed as peripheral T-cell lymphoma, NOS. Our PTCL, NOS case showed an unusual CD45 negative and CD3 dim surface expression, that raised several potential differential diagnoses like T-lymphoblastic lymphoma or blastic plasmacytoid dendritic cell neoplasm. From the methodological aspect of the case, finding small amounts of pathological cells by flow cytometry with such an unusual immunophenotype can be challenging, that requires the introduction of extended immune markers or DNA dyes into the panels and the use of alternative gating strategies.

## Data Availability

The data analyzed in this study is subject to the following licenses/restrictions: Data sets may contain personal patient data, therefore cannot be shared. Anonimized data sets are available on request at the corresponding author. Requests to access these datasets should be directed to GB, barna.gabor@semmelweis.hu.
