# Supplementary material for: Case Report: Flow cytometric differential diagnosis of a peripheral T-cell lymphoma, NOS with complete loss of CD45 and dim expression of CD3
Source: Pathol Oncol Res. 2025 May 29;31:1612095. doi: 10.3389/pore.2025.1612095 (PMC12158790; doi:10.3389/pore.2025.1612095)
Supplement: Supplementary file 1 [file DataSheet1.docx]

Supplementary material for

Case report: Flow cytometric differential diagnosis of a peripheral T-cell lymphoma, NOS with complete loss of CD45 and dim expression of CD3.

^1^Gábor Szalóki, ^1^Ágota Szepesi, ^2^Ilona Tárkányi, ^1^Ágnes Márk, ^1^Csilla Kriston, ^1^Anna Hunyadi, ^1^Réka Mózes, ^1^Gábor Barna

^1^Department of Pathology and Experimental Cancer Research, Semmelweis University, Budapest, Hungary

^2^Department of Internal Medicine and Haematology, Faculty of Medicine, Semmelweis University, Budapest, Hungary

Corresponding author: Gábor Barna, barna.gabor@semmelweis.hu

|  |  | 405 nm excitation | | | 488 nm excitation | | | | 633 nm excitation | | |
| --- | --- | --- | --- | --- | --- | --- | --- | --- | --- | --- | --- |
|  | filter | 448/45 | 528/45 | 606/36 | 527/32 | 586/42 | 700/54 | 783/56 | 660/10 | 720/30 | 783/56 |
| Tspec | fluorophore | BV421 | BV510 | BV605 | FITC |  | PC5 |  | APC |  | APC AF750 |
|  | marker | SYTO 41 | CD7 | CD45 | TRBC1 |  | CD4 |  | CD3 |  | CD8 |
|  | vendor | Invitrogen | BD | SONY | Exbio |  | BC |  | BC |  | BC |
|  | clone |  | M-T701 | HI30 | Jovi.1 |  | 13B8.2 |  | UCHT1 |  | B9.11 |
|  |  |  |  |  |  |  |  |  |  |  |  |
| IC | fluorophore | BV421 | BV510 | BV605 | FITC | PE | PC5.5 | PC7 | APC |  |  |
|  | marker | SYTO41 | CD7 | sCD3 | cyTdT | cyMPO | CD5 | CD45 | cyCD3 |  |  |
|  | vendor | Invitrogen | BD | SONY | BC | BC | BC | BC | BC |  |  |
|  | clone |  | M-T701 | UCHT1 | HT1; HT4; HT8; HT9 | CLB-MPO-1 | BL1a | J33 | UCHT1 |  |  |
|  |  |  |  |  |  |  |  |  |  |  |  |
| PDC | fluorophore | BV421 |  | BV605 | FITC | PE |  | PC7 | APC |  |  |
|  | marker | SYTO 41 |  | CD3 | CD38 | CD123 |  | CD45 | CD56 |  |  |
|  | vendor | Invitrogen |  | SONY | Dako | SONY |  | BC | BC |  |  |
|  | clone |  |  | UTCH1 | AT13/5 | 6H6 |  | J33 | N901 |  |  |
|  |  |  |  |  |  |  |  |  |  |  |  |
| LY | fluorophore | BV421 | BV510 | BV605 | FITC | PE | PC5.5 | PC7 | APC | APC AF700 | APC AF750 |
|  | marker | SYTO 41 | CD4/CD20 | CD45 | Igκ | Igλ/ CD56 | CD5 | CD10 | CD3 | CD19 | CD8 |
|  | vendor | Invitrogen | SONY | SONY | BC | BC | BC | BC | BC | BC | BC |
|  | clone |  | RPA-T4/2H7 | HI30 | ploycl. | ploycl./N901 | BL1a | ALB1 | UCHT1 | J3-119 | B9.11 |
|  |  |  |  |  |  |  |  |  |  |  |  |
| TALL1 | fluorophore | BV421 | BV510 | BV605 | FITC | PE | PERCP CY5.5 | PC7 | APC | APC AF700 | APC AF750 |
|  | marker | SYTO 41 | CD5 | CD3 | CD4 | CD99 | CD34 | CD45 | CD7 | CD2 | CD8 |
|  | vendor | Invitrogen | SONY | SONY | Dako | Exbio | SONY | BC | SONY | BC | BC |
|  | clone |  | L17F12 | UCHT1 | MT310 | 3B2/TAB | 581 | J33 | CD7-6B7 | 39C1.5 | B9.11 |
|  |  |  |  |  |  |  |  |  |  |  |  |
| TALL2 | fluorophore | BV421 | BV510 | BV605 | FITC | PE | PERCP CY5.5 | PC7 | APC |  | APC AF750 |
|  | marker | SYTO 41 | CD5 | CD3 | CD48 | CD99 | CD56 | CD45 | CD7 |  | CD8 |
|  | vendor | Invitrogen | SONY | SONY | SONY | Exbio | SONY | BC | SONY |  | BC |
|  | clone |  | L17F12 | UCHT1 | BJ40 | 3B2/TAB | 5.1H11 | J33 | CD7-6B7 |  | B9.11 |
|  |  |  |  |  |  |  |  |  |  |  |  |
| TRBC1/ TRBC2 | fluorophore | BV421 | BV510 | BV605 | FITC | PE | PC5 | PC7 | APC |  | APC AF750 |
|  | marker | SYTO 41 | CD7 | CD3 | TRBC1 | γδTCR | CD4 | CD45 | TRBC2 |  | CD8 |
|  | vendor | Invitrogen | BD | SONY | Exbio | SONY | BC | BC | BC |  | BC |
|  | clone |  | M-T701 | UCHT1 | Jovi.1 | B1 | 13B8.2 | J33 | SAM.2 |  | B9.11 |

**Supplementary Table 1.** Flow cytometric panels were used to determine the immunophenotype of the tumor cells. Invitrogen: Thermo Fisher Scientific Corp., United States; BD: Beckton Dickinson Biosciences, United States; BC: Beckman Coulter Life Sciences, United States; SONY: Sony Biotechnology, United States; Exbio: Exbio Praha, Czech Republic; Dako: Agilent Technologies, United States.


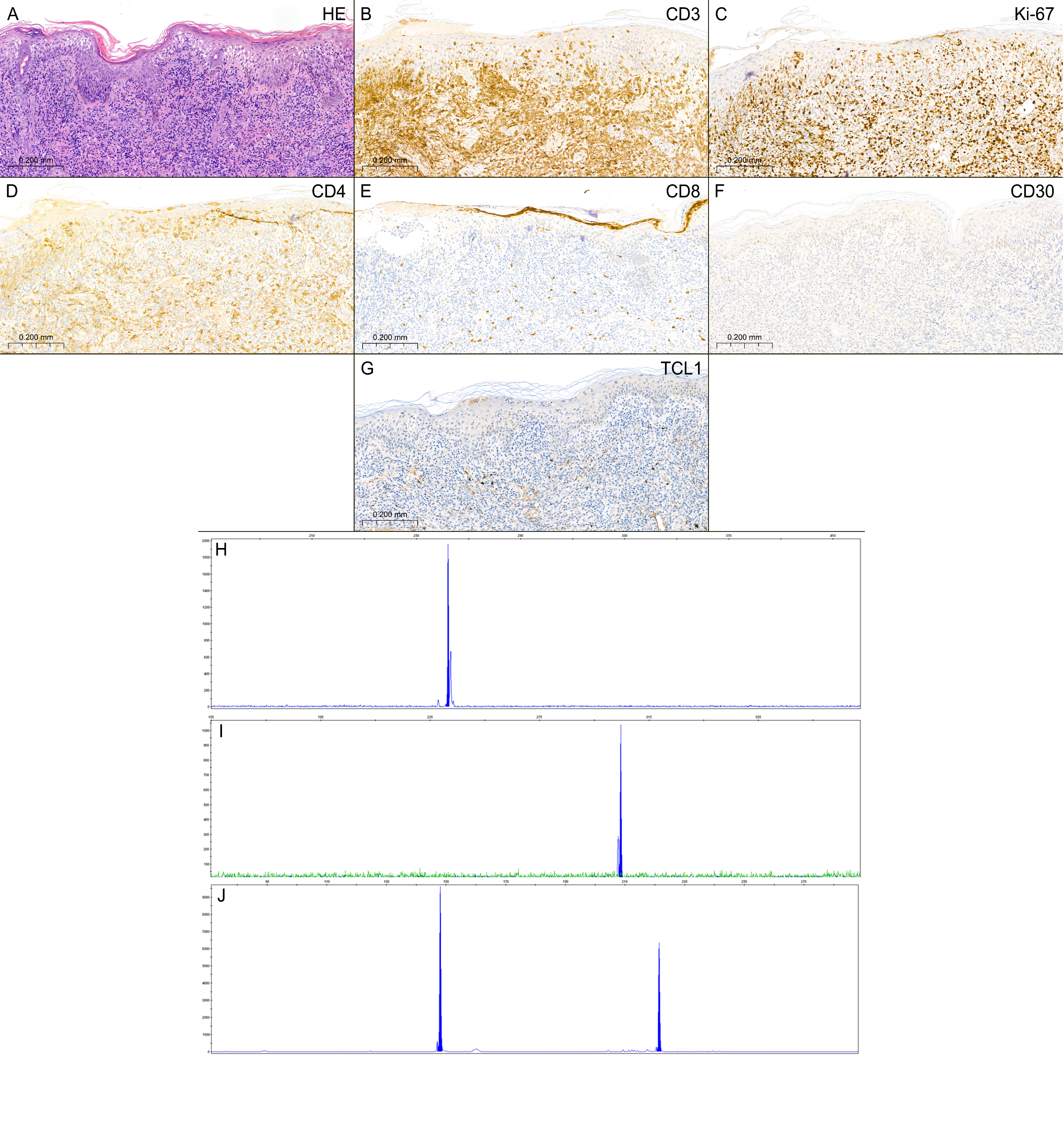


**Supplementary Figure 1.** Histology and immunohistochemistry of the patient’s skin biopsy (A-G). Hematoxylin-eosin staining (A) reveals perivascular dermal lymphoid infiltrate with mild epidermotropism. With immunohistochemistry the infiltrate was positive for CD3 (B) and CD7 (C); negative for CD8 (E), CD30 (F), and partially positive for CD4 (D). Perivascular spreading of the tumor cells is shown on G. Molecular TCR rearrangement investigation with TcRVβ – TcRJβ2 (H), TcRDβ – TcRJβ1/TcRJβ2 (I) and TcRVγfl/Vγ10 –TcRJγ (J) primer sets. All three primer sets showed monoclonal TCR rearrangement with 262 bp (H); 304 bp (I), 148 and 221 bp (J) amplicons.


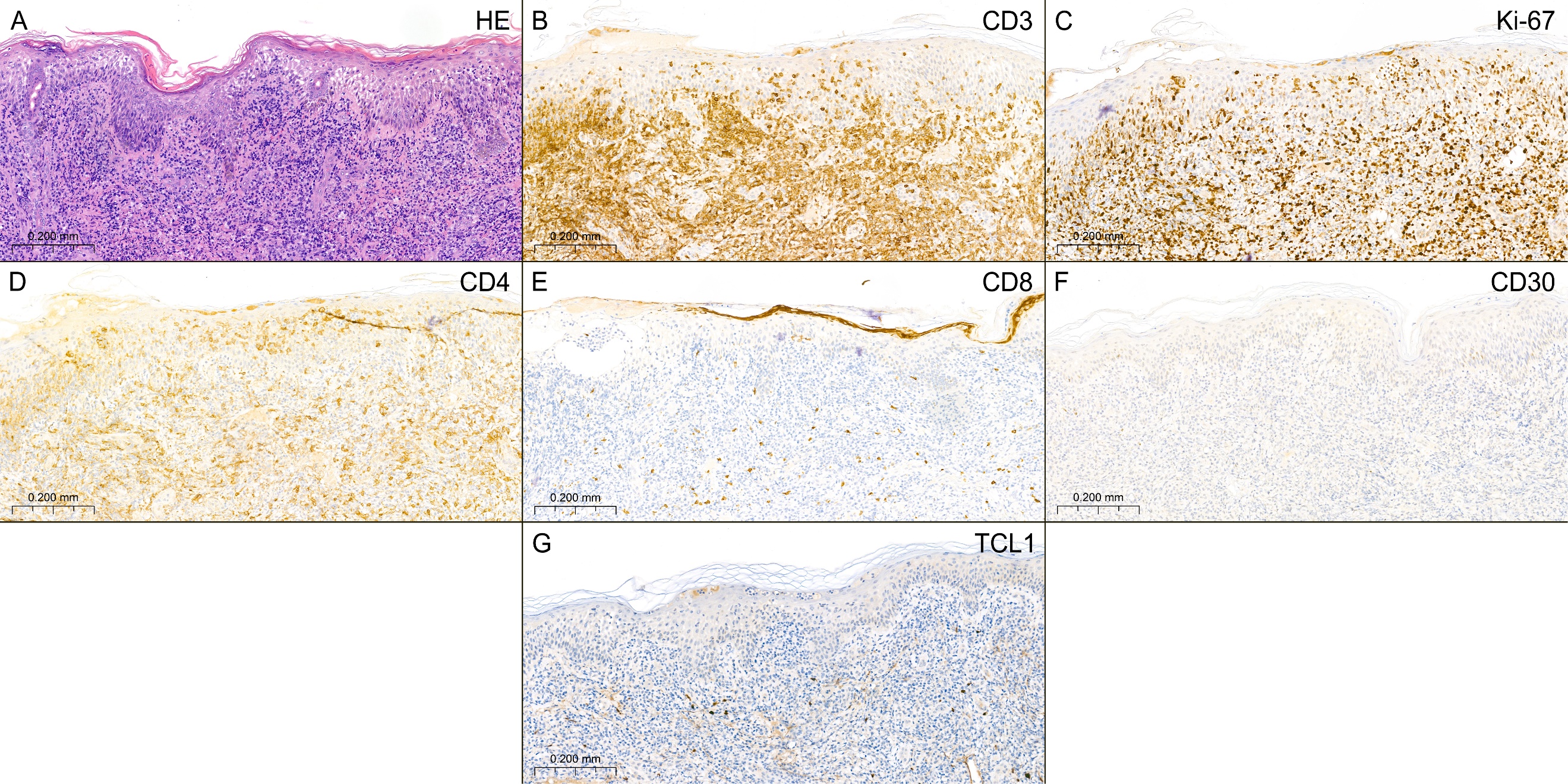


**Supplementary Figure 2.** Histology and immunohistochemistry of the patient’s skin biopsy specimen in the advanced stage of the disease. The microscopic images show diffuse dermal infiltration. The immunophenotype was unchanged, compared to the first skin biopsy (positive: CD3 (B), partially positive: CD4 (D), negative: CD8 (E), CD30 (F), TCL1 (G)). Ki-67 staining (C) showed a high, about 50% proliferation rate.


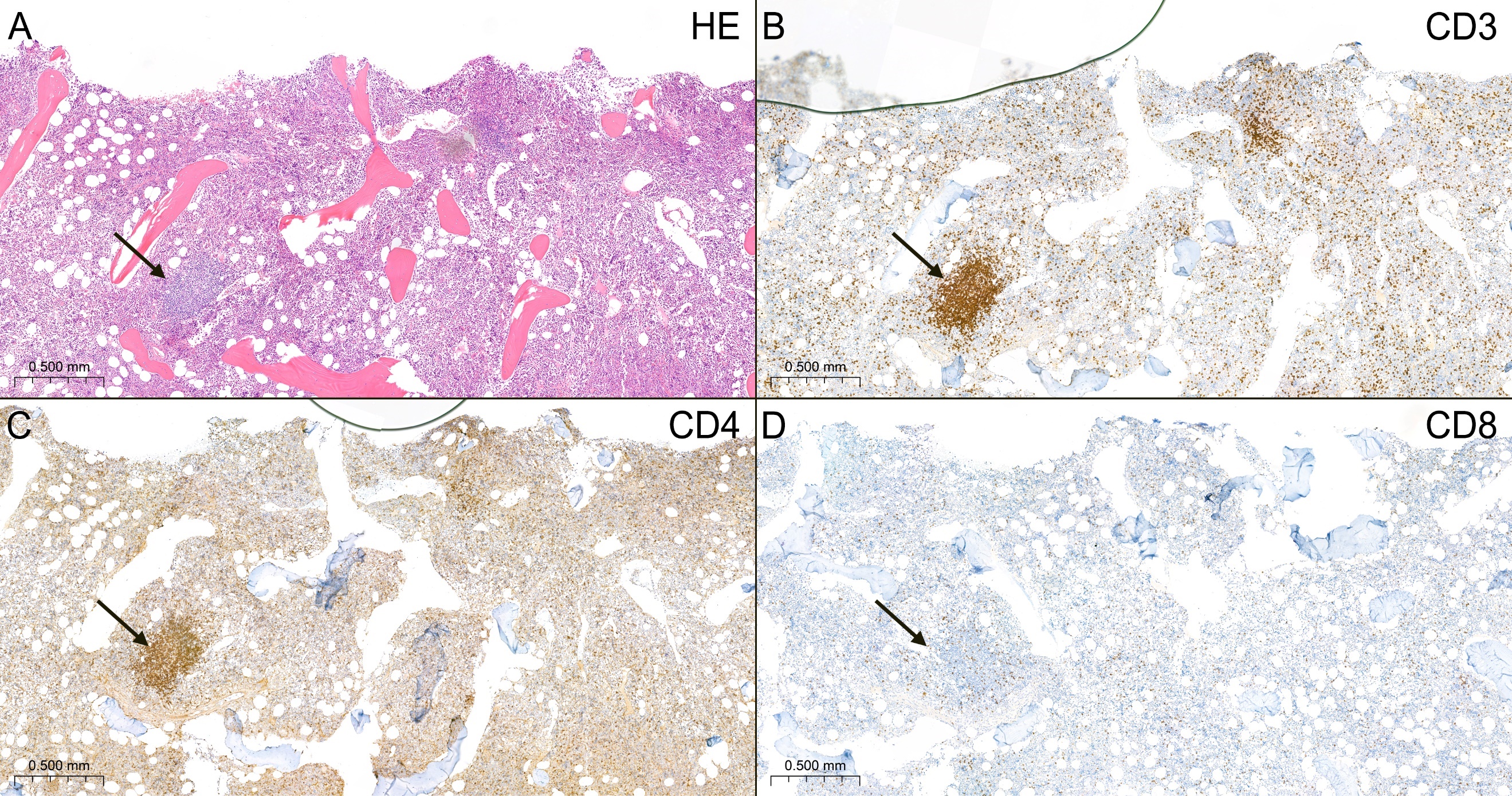


**Supplementary Figure 3.** Histology and immunohistochemistry of the patient’s bone marrow biopsy. In the whole core biopsy, there was only one nodule of T-cells (arrow), with a similar immunophenotype pattern to the pathological cells in the skin biopsy: positive for CD3 and CD4; and negative for CD8.


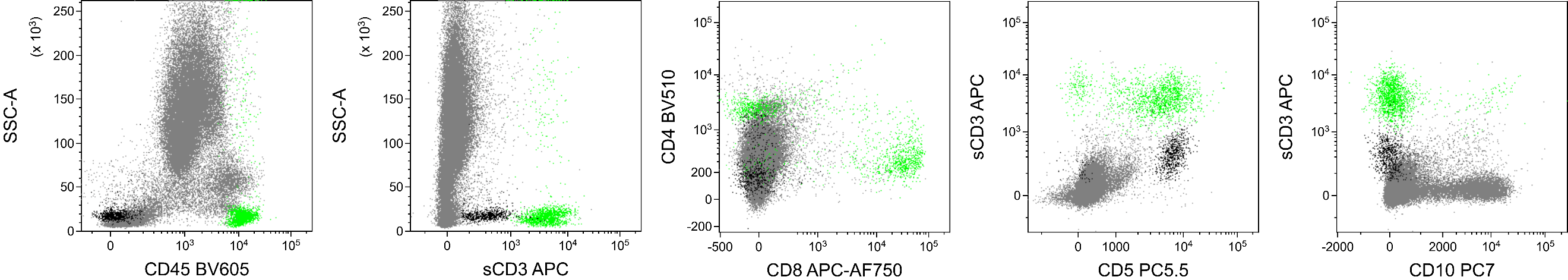


**Supplementary Figure 4**. Flow cytometric analysis of the patient's bone marrow aspirate. The sample contained 1% pathologic T-cells (black) with identical immunophenotype detected in the peripheral blood: positive: CD5; dim: sCD3; heterogeneous: CD4; negative: CD45, CD8, CD10. Normal T-lymphocytes for positive control were highlighted in green.


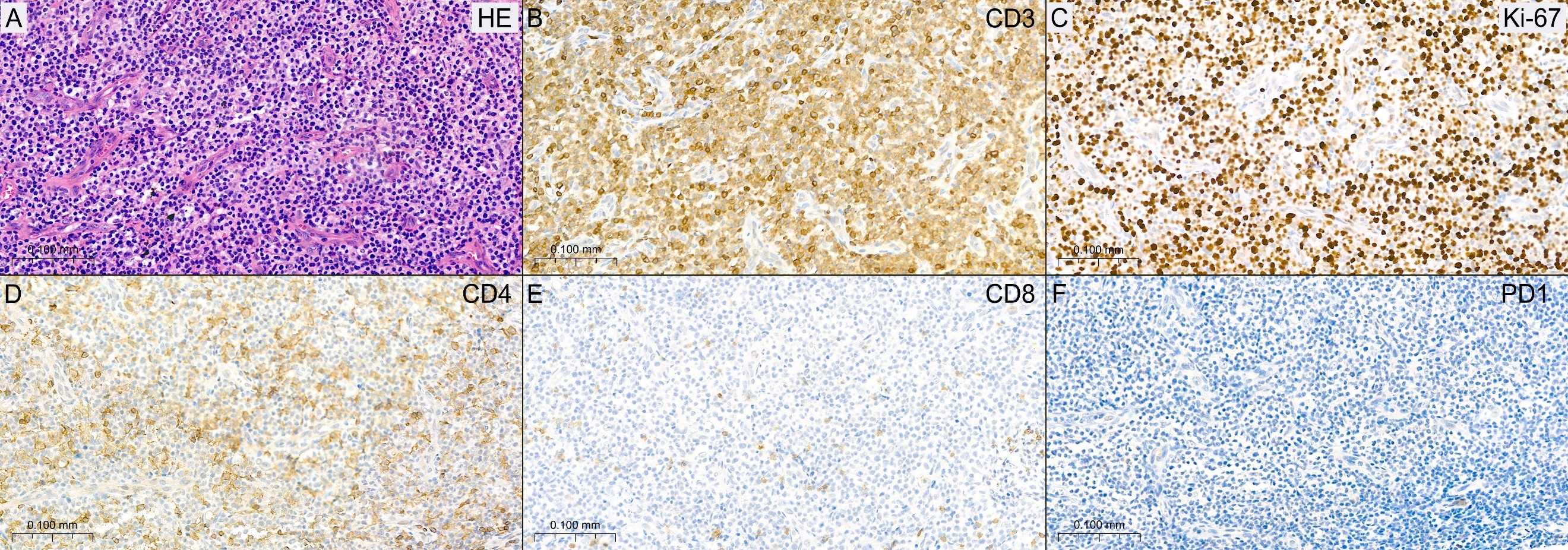


**Supplementary Figure 5.** Histology and immunohistochemistry of the patient’s inguinal lymph node. The microscopic images show a massive infiltration of pathologic T-cells, with a similar immunophenotype pattern to the pathological cells in the skin: positive for CD3 (B) and heterogeneous for CD4 (D); negative for CD8 (E). The tumor cells were negative for PD1 (F) and showed a high proliferation rate above 50% assessed with Ki-67 staining (C).


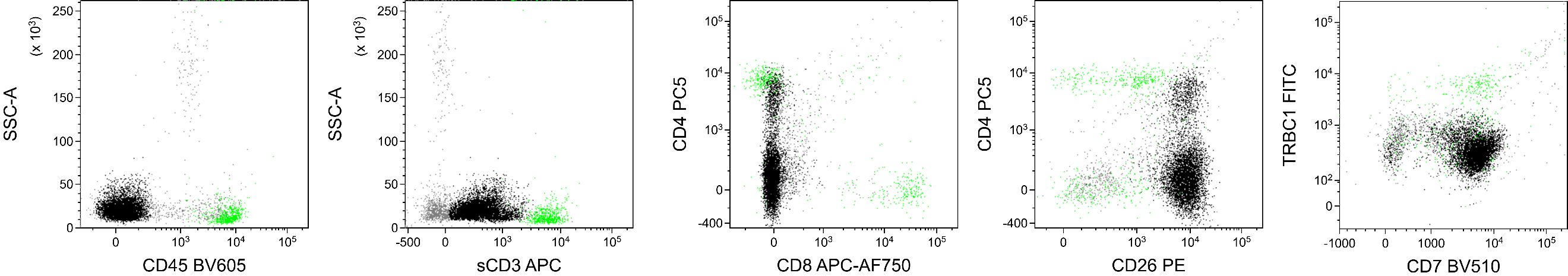


**Supplementary Figure 6.** Flow cytometric analysis of the transfer fluid (physiological saline solution) of the patient’s inguinal core biopsy specimen. The sample contained 84% pathologic T-cells (black) with the same immunophenotype as the ones in the peripheral blood: positive: CD26, CD7; dim: sCD3; heterogeneous: CD4; negative: CD45, CD8, sTRBC1. Normal T-lymphocytes for positive control were highlighted in green.
